# Lean mass sparing in resistance-trained athletes during caloric restriction: the role of resistance training volume

**DOI:** 10.1007/s00421-022-04896-5

**Published:** 2022-02-11

**Authors:** C. Roth, B. J. Schoenfeld, M. Behringer

**Affiliations:** 1 Department of Sports Medicine and Exercise Physiology, Institute of Sport Sciences, Goethe University Frankfurt, Ginnheimer Landstrasse 39, 60487 Frankfurt/Main, Germany; 2 Department of Health Sciences, CUNY Lehman College, Bronx, NY USA

**Keywords:** Protein synthesis, Protein degradation, Intracellular pathways, Weight loss, Weight training, Anabolism

## Abstract

Many sports employ caloric restriction (CR) to reduce athletes’ body mass. During these phases, resistance training (RT) volume is often reduced to accommodate recovery demands. Since RT volume is a well-known anabolic stimulus, this review investigates whether a higher training volume helps to spare lean mass during CR. A total of 15 studies met inclusion criteria. The extracted data allowed calculation of total tonnage lifted (repetitions × sets × intensity load) or weekly sets per muscle group for only 4 of the 15 studies, with RT volume being highly dependent on the examined muscle group as well as weekly training frequency per muscle group. Studies involving high RT volume programs (≥ 10 weekly sets per muscle group) revealed low-to-no (mostly female) lean mass loss. Additionally, studies increasing RT volume during CR over time appeared to demonstrate no-to-low lean mass loss when compared to studies reducing RT volume. Since data regarding RT variables applied were incomplete in most of the included studies, evidence is insufficient to conclude that a higher RT volume is better suited to spare lean mass during CR, although data seem to favor higher volumes in female athletes during CR. Moreover, the data appear to suggest that increasing RT volume during CR over time might be more effective in ameliorating CR-induced atrophy in both male and female resistance-trained athletes when compared to studies reducing RT volume. The effects of CR on lean mass sparing seem to be mediated by training experience, pre-diet volume, and energy deficit, with, on average, women tending to spare more lean mass than men. Potential explanatory mechanisms for enhanced lean mass sparing include a preserved endocrine milieu as well as heightened anabolic signaling.

## Introduction

Temporary phases of caloric restriction (CR) are typically used to reduce body mass (Gardner et al. 2018; Schwartz et al. 2017). Although, in most cases, the goal of these interventions is to reduce fat mass, lean tissue loss is often concomitantly observed as a negative side effect during prolonged CR (Ryan and Nicklas 2004; Bouchard et al. 2009; Karila et al. 2008). Weinheimer et al. (2010) concluded that, on average, 24% (dietary-only) and 11% (diet × exercise) of CR-induced weight loss are attributed to a reduction of lean tissue. Unfortunately, lean mass loss brings about additional negative consequences such as a decreased resting metabolic rate (Stiegler and Cunliffe 2006), which in turn increases the likelihood for the regain of body mass, mainly in the form of increased body fat (Maclean et al. 2011). It is therefore important to devise strategies that spare lean mass during prolonged CR interventions, which may also be of benefit to athletes who compete in sports involving body aesthetics or weight categories (e.g., bodybuilders, wrestlers, boxers, etc.). This objective, commonly referred to as *high-quality weight loss,* aims to reduce body fat while maintaining as much lean tissue as possible (Churchward-Venne et al. 2013).

Lean mass sparing is determined by the dynamic balance between muscle protein synthesis (MPS) and proteolysis (Biolo et al. 1995; Phillips et al. 1997). In this regard, CR leads to reduced responses to anabolic and anti-catabolic stimuli when compared to eucaloric conditions (e.g., leucine-induced effects on cell signaling and MPS; Gwin et al. 2020a; Pasiakos et al. 2010, 2014), potentially affects activation of proteolytic pathways via reduced insulin levels (Greenhaff et al. 2008; Tipton et al. 2018), and culminates in an increased likelihood of lean mass loss. Moreover, the rate of MPS declines as an adaptive mechanism to conserve energy, which conceivably serves as a primary reason for lean tissue loss during hypocaloric conditions (Carbone et al. 2012; Margolis et al. 2016; Miller et al. 2012). Optimal approaches for lean mass sparing during CR should therefore focus on counteracting the decline in MPS by modifying dietary and mechanical [i.e., resistance training (RT)] stimuli (Weinheimer et al. 2010; Cava et al. 2017; Carbone et al. 2019).

It is well known that a high-protein diet contributes to an elevation of mixed MPS (Pasiakos et al. 2013) and whole-body protein synthesis (Gwin et al. 2020a) as well as helping to inhibit insulin-sensitive protein breakdown (insulin-IGF-1-PI3K), which therefore partially counteracts the negative consequences of prolonged CR (Jäger et al. 2017; Hudson et al. 2020; Gwin et al. 2020b). In addition, mechanical loading, such as in RT, interacts synergistically with a high-protein diet to further elevate MPS (Phillips et al. 1997; Churchward-Venne et al. 2012). Even during CR, this combination has been shown to preserve lean mass in obese individuals (Rice et al. 1999; Longland et al. 2016) and elite-level athletes (Garthe et al. 2011). However, these findings cannot be extrapolated to resistance-trained individuals due to divergent intracellular (Moberg et al. 2020) and MPS responses (Tang et al. 2008; Damas et al. 2015), as well as blood metabolome differences (Schranner et al. 2021) between populations. To this point, a lean tissue loss of ~ 43% of the lost body mass was reported for a resistance-trained athlete undergoing CR, despite implementation of a high-protein diet and regimented RT (Kistler et al. 2014).

It is currently unknown if and how RT variables need to be adjusted to spare lean mass in resistance-trained athletes. Although different RT variables elicit different intracellular signaling responses and, thus, morphological adaptations (Toigo and Boutellier 2006), RT *volume*, in total tonnage [number of repetitions × number of sets × intensity load; kg] or simply counted as sets per muscle group per week (Baz-Valle et al. 2018; Israetel et al. 2019), might play an important role in muscular adaptations (Figueiredo et al. 2018): While several authors suggest an inverted *U*-shaped relationship between weekly volume and hypertrophy during eu- and hypercaloric conditions (Schoenfeld et al. 2019, 2017b) with higher RT volumes (up to a certain threshold) being necessary for advanced athletes to maximize hypertrophy (ACSM 2009; Krzysztofik et al. 2019), preliminary data suggest a potential positive effect of higher volume RT on lean mass sparing during periods of CR (Dudgeon et al. 2017; Mitchell et al. 2018). Contrarily, some investigators report reduced RT volume during phases of high-energy demands to accommodate recovery ability (Chaouachi et al. 2009; Meckel et al. 2008; Vargas-Molina et al. 2020; Campbell et al. 2020). Since RT volume is a well-known anabolic stimulus for muscle hypertrophy (Schoenfeld et al. 2017b), this review investigates whether higher training volumes are more appropriate for sparing lean mass during CR.

## Methods

### Inclusion criteria

The review focused on studies published in English and German language peer-reviewed journals. To meet inclusion criteria, the study had to: (1) include lean, healthy, drug-free resistance-trained individuals, (2) last at least 4 weeks, (3) investigate hypocaloric conditions (≥ 200 kcal deficit/day), (4) report pre-post data for changes in lean mass, (5) employ a high-protein diet ≥ 2.0 g/kg fat-free mass (FFM), and (6) present information about RT variables used.

### Search strategy

A systematic literature search was performed using the PubMed, MEDLINE, and SPORTDiscus databases between 1990 and December 2020 according to the Preferred Reporting Items for Systematic Reviews and Meta-Analyses (PRISMA) guidelines (Moher et al. 2009). Searches were performed using the following keywords: ‘resistance-trained’, ‘bodybuilder’, ‘bodybuilding’, ‘recreationally active’, ‘contest preparation’, ‘competition’, ‘exercise’, ‘strength training’, ‘lean mass’, ‘retention’, ‘volume’, ‘athlete’, ‘weight loss’, ‘energy restriction’, ‘energy deficit’, ‘caloric restriction’, ‘body composition’, ‘hypocaloric diet’, ‘ketogenic’, ‘time-restricted feeding’, as well as combinations of these. In addition, author names and reference lists were used for further search (Greenhalgh and Peacock 2005).

Our analysis includes initial and final body fat mass levels, protein consumption, study duration, estimated caloric deficit, RT protocol with special emphasis on RT volume used (total tonnage, sets/muscle group per week or sets/exercise), absolute and relative lean mass loss, and assessment technique. These factors were selected given their proposed role in lean tissue sparing during CR (Heymsfield et al. 2011).

### Coding of studies and methodological quality

The potentially relevant studies were perused and coded for the following criteria as per Schoenfeld et al. (2017a): (1) authors, title, and year of publication; (2) participant information, such as sample size, sex, age, and training experience. When possible, participants were categorized regarding the bodybuilding class in which they competed; in absence of this information, participants were classified based on RT experience (years of training); (3) description of the training intervention, including repetition ranges, weekly training frequency, exercises and RT volume used, with multi-joint exercises coded for the muscle group that is predominantly trained. RT volume was preferentially expressed in total tonnage (number of repetitions × number of sets × intensity load; kg) or, when not applicable, in weekly sets/muscle group. In the event that information regarding RT variables was missing, we quantified RT volume as sets/exercise. As suggested by Schoenfeld et al. (2017b), total sets per muscle group per week were categorized as follows: low (< 5), medium (5–9), or high (10 +); (4) methods of measurement were categorized as direct (magnetic resonance imaging, computerized tomography, and ultrasound) and indirect {underwater weighing (UWW), dual-energy X-ray absorptiometry (DXA), air displacement plethysmography (ADP), and bioelectrical impedance analysis (BIA)}. When direct measures were employed, we noted the specific muscle group assessed.

### PEDro scale

The PEDro scale (Maher et al. 2003) was selected to assess the methodological quality of the studies (Table 1). Consistent with previous exercise-related reviews (Schoenfeld and Grgic 2020), the first item of the scale (referring to external validity) was not taken into account for the final score as recommended in the guidelines. Furthermore, items 5, 6, and 7 were excluded as well due to the difficulty of blinding in exercise-related interventions. Thus, the maximum result was seven, categorized as follows (Schoenfeld and Grgic 2020): 6–7 = “excellent quality”; 5 = “good quality”; 4 = “moderate quality”; 0–3 = “‘poor quality”, in line with other reviews (Kümmel et al. 2016).

**Table 1:** PEDro scale (Maher et al. 2003)

Study	1	2	3	4	8	9	10	11
van der Ploeg et al. (2001)					✓	✓	✓	✓
Halliday et al. (2016)	Case study							
Hulmi et al. (2016)	✓			✓			✓	✓
Petrizzo et al. (2017)	Case study							
Rohrig et al. (2017)	Case study							
Tinsley et al. (2018)	Case study							
Vargas-Molina et al. (2020)	✓	✓	✓	✓	✓		✓	✓
Mitchell et al. (2018)	✓							✓
Pardue et al. (2017)	Case study							
Kistler et al. (2014)	Case study							
Robinson et al. (2015)	Case study							
Dudgeon et al. (2017)	✓	✓	✓		✓	✓	✓	✓
Schoenfeld et al. (2020)	Case study							
Stratton et al. (2020)	✓	✓	✓	✓			✓	✓
Campbell et al. (2020)	✓	✓	✓	✓			✓	✓

## Results

**Fig. 1:**
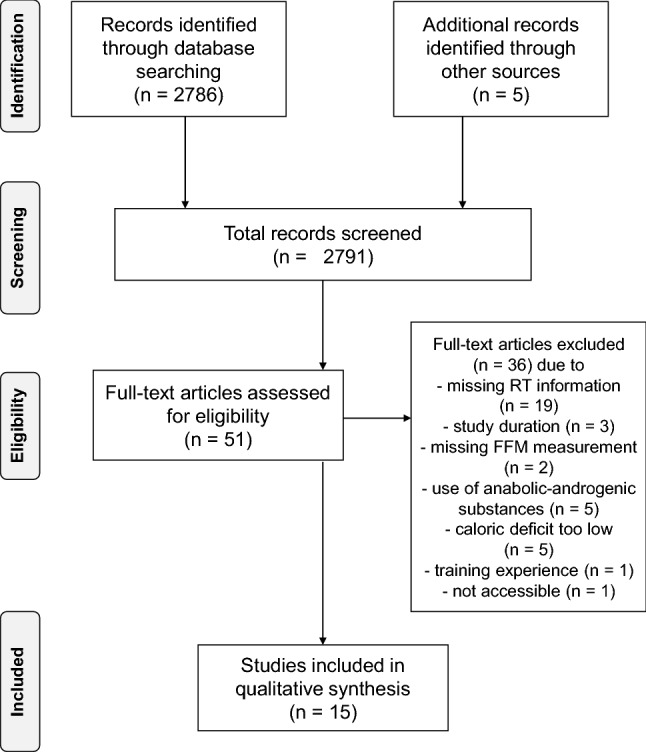
PRISMA flowchart

A total of 2791 studies were identified based on the search criteria. With respect to the studies’ abstracts, 51 of the reviewed studies were chosen to be potentially relevant for data analysis. The full texts of these articles were then screened; 36 of these studies (Greene et al. 2018; Helms et al. 2015b; Mero et al. 2010; Newton et al. 1993; Sawyer et al. 2013; Tinsley et al. 2017; Trabelsi et al. 2013, 2012; Vargas et al. 2018; Walberg-Rankin et al. 1993; Waldman et al. 2018; Kleiner et al. 1990; Bamman et al. 1993; Hickson et al. 1990; Withers et al. 1997; Wilson et al. 2017; Chatterton et al. 2017; Durguerian et al. 2016; Murphy and Koehler 2020; Gentil et al. 2017; Steen 1991; Manore et al. 1993; Too et al. 1998; Moro et al. 2016; Areta et al. 2014; Kysel et al. 2020; Philpott et al. 2019; Huovinen et al. 2015; Antonio et al. 2019; Bazyler et al. 2018; Mäestu et al. 2008, 2010; Dudgeon et al. 2016; Mettler et al. 2010; Rossow et al. 2013; Syed-Abdul et al. 2019) were excluded from analysis for various reasons. Thus, 15 studies were used for qualitative analysis. Figure 1 shows a flowchart of the literature search strategy; Table 2 summarizes the studies included for analysis.

**Table 2:** Overview of the studies meeting inclusion criteria

Authors study design population	Initial body fat [%]	Body fat end [%]	Protein [G/KG FFM]	Study duration [weeks]	Calculated mean caloric deficit/day [KCAL]	Training protocol, respectively volume used	Lean mass loss in KG (%) and assessment technique
Female RT athletes							
Van Der Ploeg et al. (2001) – Longitudinal study with isocaloric controls – Competitive female bodybuilders	18.3	12.7	–	12	~ 500	5 × per week; 6–12 repetitions/set; 3–4 sets/body part; subjects gradually increased aerobic training during the study period; *volume*: 3–4 sets/body part, no progression; 4/5 subjects reported volume (load or repetitions) drop	– 1.38 (23.8) 4C
Halliday et al. (2016) – Case study – Amateur female figure competitor	15.1	8.6	3.39	20	~ 250	4–5 × per week; frequency 2–3; 1–2 HIIT/week; aerobic exercise 1x/week; *volume*: “HV” was reported, no further information given	0.0 DXA
Hulmi et al. (2016) – Longitudinal study with isocaloric controls – Amateur female fitness competitors	14.6	7.1	3.85	M = 19.8	~ 390	4–5 × per week; frequency 1–2 (different split routines, given that the athletes continued their own workouts); participants did not significantly increase RT METh/week; Either HIIT and/or LISS was performed additionally; *volume*: RT METh/week remained constant; no further information given	+ 0.4 DXA − 1.5 (19.2) MFBIA
Petrizzo et al. (2017) – Case study – Female figure competitor	23.4	11.3	~ 4.00	32	~ 320	4–5 × with an increase to 6 × per week in the final 10 weeks; frequency 2–3; 3 sets to failure/exercise with 2–6 exercises per muscle group; Aerobic exercise and HIIT started thrice per week and increased to 4 × per week in the final 10 weeks; *volume*: progressive HVRT, 3 sets to failure/exercise with 2 (e.g., arms) to 6 (e.g., legs) exercises per muscle group per day (multiplied with a weekly frequency of 2–3)	+ 0.7 DXA
Rohrig et al. (2017) – Case study – Female physique competitor	30.5	15.9	~ 2.8–3.2	6 months	~ 420	5 × per week; frequency 2–3; one moderate volume and moderate weight (60–80% 1RM) and one lower volume and higher weight (85% + 1RM) workout; started MISS and HIIT 2 months prior to contest preparation which were gradually increased over time; *volume*: Two moderate-volume workouts/week; no further information given	+ 1.3 UWW
Tinsley et al. (2018) – Case study – Female physique competitor	20.3	12.2	~ 3.0–3.3	18	~ 270	10 distinct RT programs; 4–6 × per week; frequency 1–3; 4–20 repetitions/set; 10–20 sets per muscle group (mostly to volitional concentric failure); 25–35 sets per workout in total; interset rest intervals ~ 2 min; HIIT was emphasized in the first 4 months; thereafter, steady-state cardio was preferentially performed; during the preparation, weekly frequency of aerobic training and session duration increased gradually (up to 250–300 min per week); *volume*: self-reported HV, with 10–20 sets per muscle group (multiplied with a weekly training frequency of 2–3)	+ 1.1 4C
Vargas-Molina et al. (2020) – Longitudinal study with isocaloric controls – Resistance-trained women	29.7	29.0	2.7	8	~ 280	upper/lower split routine; 2 cycles á 4 weeks (strength, hypertrophy, endurance, recovery); 4 × per week; frequency 2; 3–25 repetitions/set; 3 sets/exercise to concentric failure (exception: strength phase, 1–2 RIR); increased training loads during the first 3 weeks of each cycle; rest between 45 s and 3 min as well as adjusted movement tempo; 72 h of recovery between each session; *volume*: 3 sets/exercise, progressively; volume (expressed in total tonnage) was reduced during deload weeks	–0.7^x^ (31.8) DXA
Male RT Athletes							
Mitchell et al. (2018) – Longitudinal study without controls – Male resistance-trained athletes	10.5	6.7	3.42–3.74	16	~ 260	Total volume (total tonnage) was used progressively from PRE16 to week 8 (PRE8; Δ + 11.800 kg^x^) and was dropped afterward to week 1 (PRE1; Δ -27.700 kg^x^); *volume*: progressive in terms of total tonnage (week 16 to week 8), followed by a volume drop between week 8 and week 1 – no further information given	– 0.5 (12.2) DXA
Pardue et al. (2017) – Case study – Male resistance-trained athlete	13.8 DXA 13.4 ADP	5.1 DXA 9.6 ADP	3.36–4.14	8 months	~ 280	5–6 × per week; frequency at least 2; 4–25 repetitions/set; 3–4 sets/exercise with up to 3 exercises per muscle group (based on the provided sample plan); Cardio (combination of HIIT and MISS) was gradually increased to support weight loss (finally reaching 2 × 20 min HIIT and 4 × 30 min MISS per week); *volume*: 3–4 sets/exercise with up to 3 exercises per muscle group (multiplied with a weekly training frequency of 2)	– 0.9 (9.8) DXA – 5.0 (54.9) ADP
Kistler et al. (2014) – Case study – Male resistance-trained athlete	17.5	7.4	3.3–3.6	26	~ 580	5 × per week; frequency 2; DUP; 3–15 repetitions/set; 3–8 exercises/workout*; 20–25 sets/workout* throughout the study; 2 × 40 min HIIT (increased at the end of the preparation phase) and additional aerobic exercise/week systematically increased to maintain constant weight loss; *volume*: 20–25 sets/workout; no further information given	– 6.6 (43.1) DXA
Robinson et al. (2015) – Case study – Male resistance-trained athlete	14.0	7.2	2.6–3.4	14	882 ± 433	4 × per week; frequency 2; 8–10 repetitions/set; 4–5 sets/exercise; 6–8 exercises/workout; HIIT: 1x/week, 10 × 10–15 s; LISS: 40 min incline walk on the treadmill 2x/week in weeks 8–10; increased to 5x/week in weeks 11–14; *volume*: 4–5 sets/exercise	–5.0 (42.7) skinfolds
Dudgeon et al. (2017) – Longitudinal study – + Whey group Male resistance-trained athletes	-	-	35–40% + 56 g Whey	8	~ 320	4 × per week; frequency 1; 3–4 sets/exercise, starting with 4–5 repetitions per set; progressively (up to 10–11 repetitions in weeks 7 and 8), 2 repetitions were added after each week; 2 min interset rest; supervised; *volume*: 3–4 sets/exercise; progressively	0.0 UWW
Dudgeon et al. (2017) + Carb group Male resistance-trained athletes	-	-	35–40%	8	~ 290	See above	− 0.9 (39.1) UWW
Schoenfeld et al. (2020) – Case study – Amateur male bodybuilder	9.5	5.6	~ 3.0	8 months	~ 320	3–7 × per week; whole-body routines; frequency mostly 5–6; repetitions ranged between 6–15 (R: 3–30); 3–4 sets/exercise (R: 1–10) with shoulders, arms or abdominals being trained with fewer exercises; 10–14 exercises/session [(Mdn); R: 2–23]; 30 min of daily walks; *volume*: self-reported HV, 3–4 sets/exercise (R: 1–10)	− 5.8 (54.0) MFBIA quadriceps MT remained preserved until month 5 and then declined by ~ 9% skinfolds − 6.0 (58.3)
Stratton et al. (2020) – Longitudinal study – If protocol Recreationally active athletes	19.9	18.3	~ 2.3	4	~ 300	supervised whole-body routines; 3 × per week; frequency 3; 3–8 repetitions/set; 2–4 sets/exercise; interset rest 1–2 min; DUP; *volume*: 2–4 sets/exercise; load increased progressively in a weekly fashion	+ 2.7 cm^2^ vastus lateralis CSA + 0.8 cm^2^ biceps brachii CSA US 4C (data not presented)
Stratton et al. (2020) Continuous dieting Recreationally active athletes	18.9	17.4	~ 2.3	4	~ 350	See above	+ 1.5 cm^2^ vastus lateralis CSA + 0.7 cm^2^ biceps brachii CSA US 4C (data not presented)
Mixed-sex groups							
Campbell et al. (2020) – Longitudinal study – Intermittent energy restriction Resistance-trained individuals	21.6	18.8	2.16	7	~ 460	Supervised upper and lower body split except week 4 which only consisted of 2 workouts; frequency 2; Upper body: 6 exercises/session; sets included 3 sets progressing to 4 sets with 6–8 and 8–10 repetitions; Lower body: 5 exercises/session; sets included 3 sets progressing to 4 sets with 6–8 and 8–10 repetitions except for calf raises which were trained in the range of 12–15 repetitions; Aerobic exercise was performed twice a week at a low to moderate intensity with a duration of 30 min *volume*: 3–4 sets/exercise, progressively, with reduced training volume in week 4	– 0.4 (12.5%) MFBIA
Campbell et al. (2020) Continuous dieting Resistance-trained individuals	20.6	18.6	2.12	7	~ 510	See above	– 1.3 (36.1%) MFBIA

Legend: *4C*  4-compartment model, *ADP* air displacement plethysmography, *CSA*  cross-sectional area, *DUP* daily undulating periodization, *DXA* dual-energy X-ray absorptiometry, *FFM* fat-free mass, *HIIT* high-intensity interval training, *HV *high volume, *IF* intermittent fasting, *LISS* low-intensity steady state, *M*  mean value, *Mdn* median, *METh/week* Metabolic equivalent hours/week, *MFBIA*  multifrequency bioelectrical impedance analysis, *MISS* moderate-intensity steady state, *MT*  muscle thickness, *R* range, *RIR*  repetitions in reserve, *RM*  Repetition Maximum, *RT*  resistance training, *US* ultrasound, *UWW *underwater weighing, *indicates data from individual email correspondence with the authors, ^x^ indicates that statistical significance was not reached

### Sex distribution, study design, and intervention period

Seven studies recruited female participants. Of these 7 studies, 3 employed a longitudinal design (van der Ploeg et al. 2001; Hulmi et al. 2016; Vargas-Molina et al. 2020), while the remaining 4 were case studies (Halliday et al. 2016; Petrizzo et al. 2017; Rohrig et al. 2017; Tinsley et al. 2018). Seven studies recruited male participants. Out of these 7 studies, 3 studies employed a longitudinal design (Dudgeon et al. 2017; Stratton et al. 2020), with one study providing no control group (Mitchell et al. 2018): the other 4 studies were case studies (Pardue et al. 2017; Kistler et al. 2014; Robinson et al. 2015; Schoenfeld et al. 2020). One study (Campbell et al. 2020) used mixed-sex groups. The average study duration was 18.19 weeks (Mdn = 18 weeks) and ranged between 4 weeks (Stratton et al. 2020) and 8 months (Schoenfeld et al. 2020; Pardue et al. 2017).

### Participants’ characteristics

The studies finally encompassed a total of 129 participants, consisting of 60 female and 69 male participants. Across the studies, 48 participants withdrew, with 5 studies reporting the reasons for withdrawal (Campbell et al. 2020; Mitchell et al. 2018; Stratton et al. 2020; Hulmi et al. 2016; Vargas-Molina et al. 2020). A majority of studies employed resistance-trained athletes except for Stratton et al. (2020), who characterized participants as recreationally trained. On average, mean training experience equated to 6.02 years (Mdn = 5.5 years) and ranged between “at least 6 months” (Stratton et al. 2020) to 10 years (Schoenfeld et al. 2020; Kistler et al. 2014). Mean age was 25.9 years ranging from 21 (Pardue et al. 2017; Robinson et al. 2015) to 35.3 years (van der Ploeg et al. 2001). Initial body fat averaged 21.7% [ranging from 14.6% (Hulmi et al. 2016) to 30.5% (Rohrig et al. 2017)] and 14.9% [ranging from 9.5% (Schoenfeld et al. 2020) to 19.9% (Stratton et al. 2020)] in females and males, respectively; ∆ body fat loss averaged  – 7.9% in females and  – 5.2% in males in total.

### Estimated caloric deficit, diet differences, and protein consumption

Based on the amount of weight lost, the estimated energy deficit per day ranged between  – 250 kcal (Halliday et al. 2016) and  – 882 kcal per day (Robinson et al. 2015) with a mean deficit of  – 347 kcal and  – 398 kcal per day in females and males, respectively. Most of the studies reported a gradual decrease in energy intake during the trials. One study (Campbell et al. 2020) employed an intermittent dieting protocol [5 days dieting ( – 35% reduction from maintenance caloric needs) followed by a 2-day diet break (consumption at maintenance caloric needs) and included a continuous dieting group as controls ( – 25%)]. While most of the studies were carried out using a low-fat diet, 3 studies employed a carbohydrate-cycling protocol (Halliday et al. 2016; Kistler et al. 2014; Pardue et al. 2017) and 1 study used a ketogenic diet protocol (Vargas-Molina et al. 2020). Mean protein consumption was 3.35 g/kg FFM in females and 3.06 g/kg FFM in males, and ranged between 2.12 g/kg FFM (Campbell et al. 2020) and 4.00 g/kg FFM (Petrizzo et al. 2017).

### Exercise protocols, volume quantification, lean mass change, and assessment techniques

Eleven out of 15 studies used concurrent aerobic training (high-intensity interval, moderate-intensity, or low-intensity steady-state training), which was typically (van der Ploeg et al. 2001; Petrizzo et al. 2017; Rohrig et al. 2017; Tinsley et al. 2018; Kistler et al. 2014; Robinson et al. 2015; Hulmi et al. 2016; Pardue et al. 2017), but not always (Campbell et al. 2020; Halliday et al. 2016; Schoenfeld et al. 2020), increased to support gradual weight loss. RT per week varied between 2 and 7 days. Most studies employed 2 RT sessions per muscle group; however, this ranged between 1 (Dudgeon et al. 2017) and 7 (Schoenfeld et al. 2020) days per week. Repetitions ranged from 3 to 30 across trials with 3 studies employing RT to concentric failure (Petrizzo et al. 2017; Tinsley et al. 2018; Vargas-Molina et al. 2020) and 3 studies reporting daily undulating periodized loading schemes (Kistler et al. 2014; Stratton et al. 2020; Rohrig et al. 2017). Interset rest intervals ranged between 45 s and 3 min, but were only reported in 4 studies (Tinsley et al. 2018; Vargas-Molina et al. 2020; Dudgeon et al. 2017; Stratton et al. 2020).

Only one of the included studies (Mitchell et al. 2018) provided volume quantification in total tonnage lifted (repetitions × sets × intensity load). Weekly sets per muscle group were reported in 3 studies (Petrizzo et al. 2017; Tinsley et al. 2018; Pardue et al. 2017). Volume configurations were highly dependent on the examined muscle group, as well as on the number of weekly training sessions for the same muscle group. Overall, volume ranged from 10 sets per muscle group per week (e.g., arms; Petrizzo et al. 2017; Tinsley et al. 2018) to > 20 (Pardue et al. 2017) and > 30 sets/muscle group per week (mostly legs; Petrizzo et al. 2017). In the remaining studies, volume was either depicted as sets/exercise (van der Ploeg et al. 2001; Vargas-Molina et al. 2020; Robinson et al. 2015; Dudgeon et al. 2017; Schoenfeld et al. 2020; Stratton et al. 2020; Campbell et al. 2020), ranging between 1 and 10 sets/exercise, or could not be calculated adequately (Halliday et al. 2016; Hulmi et al. 2016; Rohrig et al. 2017; Kistler et al. 2014). Notably, 1 study (Halliday et al. 2016) referenced their RT protocol as high-volume without providing any information about RT volume. Finally, 3 of the 15 studies were carried out under direct supervision (Campbell et al. 2020; Dudgeon et al. 2017; Stratton et al. 2020).

Five studies increased RT volume over time either via sets/exercise or by increasing the weight lifted (Petrizzo et al. 2017; Vargas-Molina et al. 2020; Dudgeon et al. 2017; Stratton et al. 2020; Campbell et al. 2020), with 1 study mentioning RT changes in METh/week (Hulmi et al. 2016). Three studies reduced RT volume (van der Ploeg et al. 2001; Vargas-Molina et al. 2020; Campbell et al. 2020), whereas 1 study reported an increase followed by a decrease in total tonnage lifted during the course of bodybuilding contest preparation (Mitchell et al. 2018).

**Fig. 2:**
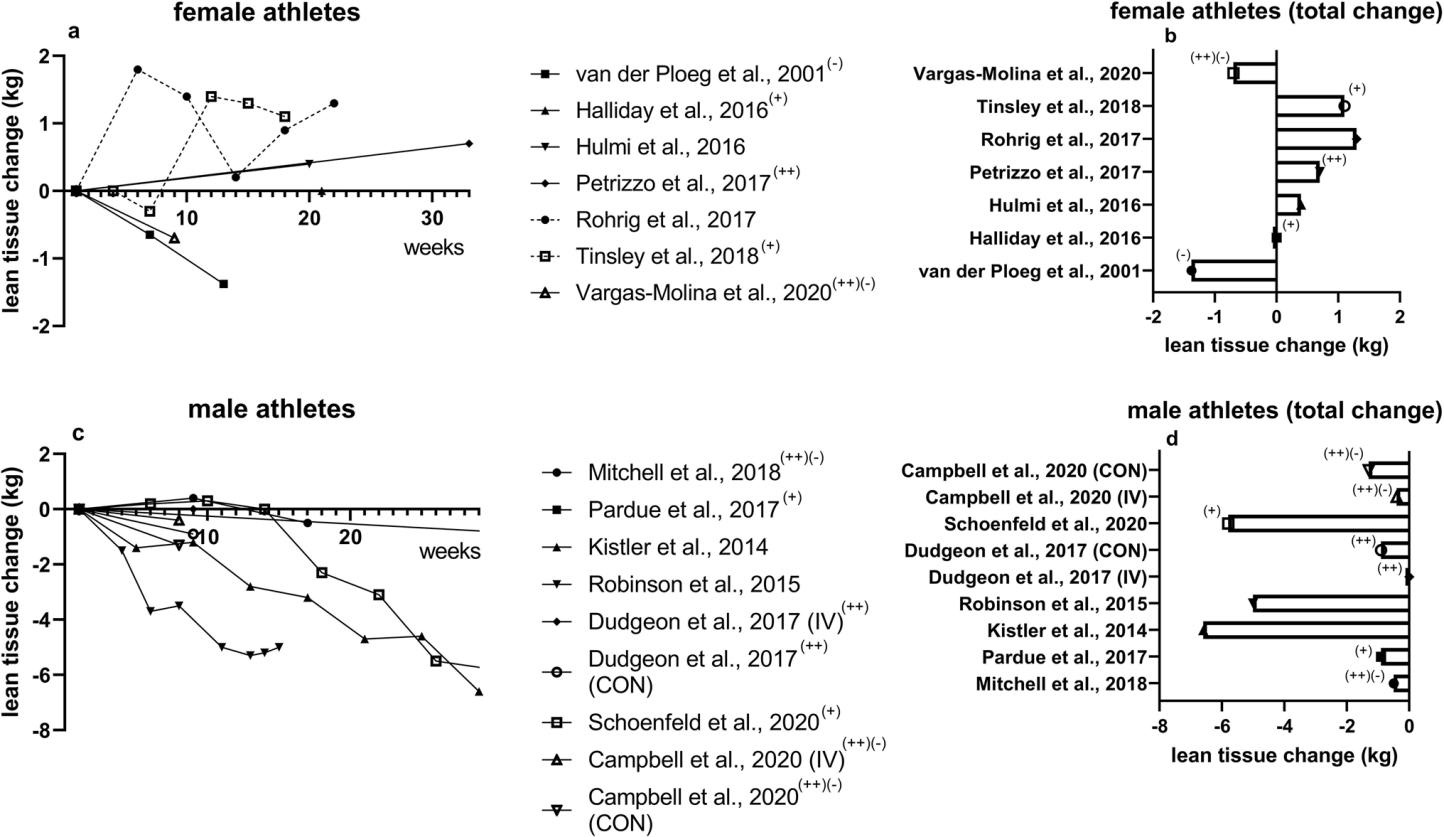
Time course of lean tissue changes during CR in kilograms. Longitudinal depiction of (**a**) female and (**c**) male athletes as well as absolute visualization of (**b**) female and (**d**) male athletes. (+) indicates (self-reported) high-volume RT; (++) indicates progressive overload over time, (-) indicates reduced volume; without symbols means that volume tendency could not be specified; Stratton et al. 2020 was not included due to missing whole-body lean mass data. *CON* control group,* IV* intervention group

In general, average body mass loss was  – 4.8 kg and  – 5.0 kg in females and males, respectively. Lean mass changes varied between + 1.3 kg and  – 6.6 kg, with a relative lean tissue loss up to approximately 54% of the total loss in body mass (Schoenfeld et al. 2020). When divided by sex, 2 of 7 studies in females and 5 of 7 studies in males reported lean tissue loss during dieting, suggesting a significant influence of sex on lean tissue change during CR. On average, females gained 0.2 kg lean tissue during dieting with 1 study (Hulmi et al. 2016) reporting contradictory results between DXA and multifrequency BIA. Except for 2 studies (van der Ploeg et al. 2001; Vargas-Molina et al. 2020), the studies using female participants reported muscle mass maintenance (Halliday et al. 2016) or even an increase in lean tissue (+ 1.03 kg on average) (Petrizzo et al. 2017; Rohrig et al. 2017; Tinsley et al. 2018). Except for Rohrig et al. (2017), who reported moderate volume workouts, the latter mentioned studies labeled themselves as high-volume or increased RT volume over time (CR ranging from -270 kcal/day to  – 320 kcal/day) and can be categorized as high-volume according to our a priori classification criteria. Contrarily, studies reporting decreased RT volume (Vargas-Molina et al. 2020; van der Ploeg et al. 2001) observed lean tissue losses ( – 1.04 kg on average; CR ranging from  – 280 kcal/day to  – 500 kcal/day). The time course of absolute lean tissue changes for men and women is presented in Fig. 2.

On average, the lean tissue loss was  – 2.81 kg in males (Mdn =  – 0.9 kg) with a relative mean loss of 28.7% of the loss in total body mass (Mdn = 39.1%). While absolute lean mass loss ranged from −0.5 kg to −6.6 kg, relative losses ranged between 12% and 54% of the weight lost, with 1 study (Pardue et al. 2017) reporting contradictory results between DXA and ADP. Except for 1 study that reported no lean tissue loss in the + WHEY intervention group (Dudgeon et al. 2017) and another study reporting increases in cross-sectional area (CSA) (Stratton et al. 2020), the remaining 5 studies observed lean tissue losses (Robinson et al. 2015; Schoenfeld et al. 2020; Kistler et al. 2014; Pardue et al. 2017; Mitchell et al. 2018). When evaluating the relationship between lean tissue changes and RT volume, studies that reported no loss (Dudgeon et al. 2017; Stratton et al. 2020) or even an increase in lean mass after 8 weeks of CR (Mitchell et al. 2018), all increased RT volume over time (CR ranging from  – 260 kcal/day to  – 350 kcal/day). Contrarily, when RT volume was reduced, a loss of lean mass was reported (Mitchell et al. 2018). Overall, most of the studies assessed body composition via DXA (Kistler et al. 2014; Halliday et al. 2016; Hulmi et al. 2016; Petrizzo et al. 2017; Vargas-Molina et al. 2020; Mitchell et al. 2018; Pardue et al. 2017), followed by multifrequency BIA (Hulmi et al. 2016; Schoenfeld et al. 2020; Campbell et al. 2020), skinfold measures (Hulmi et al. 2016; Robinson et al. 2015; Schoenfeld et al. 2020), 4C models (van der Ploeg et al. 2001; Tinsley et al. 2018), UWW (Rohrig et al. 2017; Dudgeon et al. 2017), and ADP (Pardue et al. 2017). Three studies used ultrasound as a direct measure (Hulmi et al. 2016; Schoenfeld et al. 2020; Stratton et al. 2020).

## Discussion

The purpose of this review was to assess whether higher RT volumes help to spare lean mass during CR. Volume configurations were highly dependent on the examined muscle group as well as on the number of weekly training sessions for the same muscle group. According to the volume classification used in this review, 3 studies could be categorized as high-volume, ranging from 10 to > 30 weekly sets per muscle group, which revealed low-to-no (mostly female) lean mass loss. Volume quantification in total tonnage was only provided by 1 study (Mitchell et al. 2018), with the authors reporting an increase in lean mass after increasing the total tonnage lifted (repetitions × sets × intensity load). Unfortunately, the provided data allowed calculation of total tonnage lifted or weekly sets per muscle group for only 4 of the 15 studies. Therefore, due to incomplete data regarding RT variables applied, there is insufficient evidence to conclude that a higher RT volume is advantageous for sparing lean mass during CR, although the data do seem to favor high-volume RT in female athletes under these circumstances. Longitudinal studies using male participants under CR are less conclusive, with 1 study reporting high lean mass loss despite being referenced as high-volume by the authors (Schoenfeld et al. 2020). Moreover, the data appear to suggest that progressively increasing RT volume over time during CR may be more effective in ameliorating CR-induced atrophy in both female and male resistance-trained athletes as opposed to reducing RT volume. Our conclusion appears to be supported by endocrine (Schoenfeld et al. 2020; Stratton et al. 2020; Rossetti et al. 2017) and intracellular findings (Areta et al. 2014; Pasiakos et al. 2013; Hornberger 2011), underpinning potential explanations for increased lean mass sparing. Our findings also indicate that, on average, women tend to spare more lean mass than men during CR.

### Female resistance-trained athletes

Seven CR studies with female participants met our inclusion criteria (Halliday et al. 2016; Hulmi et al. 2016; Petrizzo et al. 2017; Rohrig et al. 2017; Tinsley et al. 2018; van der Ploeg et al. 2001; Vargas-Molina et al. 2020). Except for three studies (van der Ploeg et al. 2001; Hulmi et al. 2016; Vargas-Molina et al. 2020), the remaining studies reported muscle mass maintenance (Halliday et al. 2016) or an increase in lean mass (Petrizzo et al. 2017; Rohrig et al. 2017; Tinsley et al. 2018). When evaluating the relationship between lean tissue changes and RT volume, the latter mentioned studies either self-reported their volume as moderate without providing set numbers (Rohrig et al. 2017), increased RT volume over time (Petrizzo et al. 2017), or met our established criteria as a high-volume protocol (Petrizzo et al. 2017; Tinsley et al. 2018). Conversely, studies reporting decreased RT volume (van der Ploeg et al. 2001; Vargas-Molina et al. 2020) tended to observe lean tissue loss.

For instance, van der Ploeg et al. (2001) reported that participants lost -1.38 kg lean mass on average (23.8% of the lost mass). Although the study did not report protein intake, volume (in terms of load or repetitions) was reduced by 4/5 participants during the study. Since the remaining studies did not report any changes in the RT protocol (Halliday et al. 2016; Rohrig et al. 2017), increased volume (Petrizzo et al. 2017), or steadily changed RT programing to induce novel stimuli (Tinsley et al. 2018), it is plausible that the reduced volume contributed to the lean mass loss. This is in accordance with other research reporting a similar lean mass loss and decreased volume in 2 of 8 weeks (Vargas-Molina et al. 2020).

Except for one study (Halliday et al. 2016) that reported lean mass maintenance, the remaining studies (Petrizzo et al. 2017; Rohrig et al. 2017; Tinsley et al. 2018) found that female athletes gained lean mass during CR. Unfortunately, Rohrig et al. (2017) only reported information on two moderate-volume and two high-intensity workouts without providing further details (lean mass gain + 1.3 kg), thus precluding in depth analysis. Other studies provided more detailed information about exercise and nutritional variables (Petrizzo et al. 2017; Tinsley et al. 2018): For instance, Petrizzo et al. (2017) stated that their participant ingested ~ 4.00 g/kg FFM protein/day on average and completed high-volume RT during the course of the study (lean mass gain + 0.7 kg). Contest preparation started with RT performed 4–5 × per week and progressively increased to 6x/week, while RT volume per session remained the same. In every workout, the athlete trained with 2–6 exercises per muscle group with 3 sets to momentary muscle failure per exercise. Although it is unclear whether RT volume was increased over time in the study by Tinsley et al. (2018), the high-volume RT protocol (4–6 times/week on average with 10–20 sets per muscle group) led to a significant increase in lean mass (+ 1.1 kg). It is noteworthy that the majority of studies employed protein intakes at the upper limits of what is recommended for weight loss phases (Roberts et al. 2020; Helms et al. 2014). Since a high-protein intake appears to attenuate stress and fatigue compared to a moderate-protein intake during CR (Helms et al. 2015b), it is plausible that the markedly high-protein intake seen in this review might have allowed a non-perturbed RT performance, potentially helping to indirectly spare lean mass. More research is warranted to elucidate the interaction between protein intake and RT performance (e.g., referring to volume tolerance, strength change, stress, and fatigue accumulation) during CR.

Although preliminary evidence seems to suggest that high-volume RT leads to an increase in lean mass during CR in resistance-trained female athletes (Petrizzo et al. 2017; Tinsley et al. 2018), incomplete data hamper the strength of relationship between variables. However, since studies that cut back RT volume reported atrophic effects, increasing volume over time during CR appears to be a promising approach to achieve high-quality weight loss in female athletes. This is in line with the proposed inverted U-shaped relationship of hypertrophy during eucaloric conditions, with RT volume appearing to elicit anabolism in a dose-dependent fashion (Schoenfeld et al. 2017b; Figueiredo et al. 2018), and leads us to speculate that similar anabolic effects may occur during CR as well in a young, female population (Petrizzo et al. 2017; Tinsley et al. 2018). This hypothesis should be tested in well-controlled studies to better infer causality. Moreover, future research should provide a detailed description of how RT variables were applied, given the incomplete data regarding RT variables seen in this review. In this context, we recommend providing workout details with the variables identified by Toigo and Boutellier (2006) that have all been shown to affect the adaptation response following repeated bouts of RT.

### Male resistance-trained athletes

Seven studies using male participants (Mitchell et al. 2018; Pardue et al. 2017; Kistler et al. 2014; Dudgeon et al. 2017; Stratton et al. 2020; Schoenfeld et al. 2020; Robinson et al. 2015) and 1 study using mixed-sex groups (Campbell et al. 2020) met inclusion criteria of our review. Except for 2 studies (Dudgeon et al. 2017; Stratton et al. 2020), the remaining 6 studies reported a wide range of lean tissue losses. When evaluating the relationship between lean tissue changes and RT volume in this population, studies reporting low (Dudgeon et al. 2017) [+ CARB group] to no loss (Dudgeon et al. 2017) [+ WHEY group] or even an increase (Stratton et al. 2020) in CSA, involved an increased RT volume over time. These findings are supported by a study that increased volume during the first few weeks of the CR and then reduced RT volume during the final weeks (Mitchell et al. 2018).

Dudgeon et al. (2017) recruited RT athletes with at least 2 years of RT experience and compared lean mass changes when consuming higher versus lower protein diets during CR. The supervised RT protocol included 3–4 sets/exercise with steady RT volume increases throughout the course of the study. The higher protein group fully retained initial lean mass with the control group slightly losing lean mass [−0.9 kg (39.1%)]. Moreover, the study by Stratton et al. (2020) involved recreationally active athletes with “at least 6 months” of RT experience. Participants performed a supervised RT protocol that progressively increased load over time. At study’s end, ultrasound measurements revealed a significant increase in m. vastus lateralis and m. biceps brachii CSA. These findings suggest that increasing RT volume could possibly elicit lean tissue accretion in recreationally trained athletes under conditions of CR. The results are in accordance with other studies (Garthe et al. 2011; Longland et al. 2016; Barakat et al. 2020), suggestive of a mediating effect of RT experience on lean mass sparing during CR. Consequently, findings using RT beginners or novice athletes cannot be extrapolated to populations who are chronically adapted to RT. Beginners or novice athletes are hypersensitized to RT-induced stimuli, which probably leads to a better preservation or, perhaps even an accretion of lean mass during hypocaloric conditions (Garthe et al. 2011; Longland et al. 2016).

Current data suggest that increasing RT volume over time might have beneficial effects on sparing lean mass during CR in resistance-trained males, as well. This hypothesis is supported by longitudinal data showing an increase in lean mass (0.4 kg) during the first 8 weeks of contest preparation (~ -260 kcal/day) of resistance-trained men (Mitchell et al. 2018). The athletes trained progressively (total tonnage per week) between week 16 (PRE16) and week 8 (PRE8) (volume increased from 82,500 kg to 94,300 kg). However, when the athletes reduced total volume (94,300 kg to 66,600 kg) from week 8 (PRE8) to week 1 (PRE1), a lean mass loss of -0.5 kg on average was observed. Similar results were found in the case study by Schoenfeld et al. (2020). Although volume cannot be calculated retrospectively, a high RT volume (range: 1–10 sets/exercise performed 6–7 days/week) was reported over the course of the study with no alterations in lean mass seen during the first 3 months of contest preparation as indicated by multifrequency BIA. This interpretation is supported by the complementary ultrasound measurements (muscle thickness) revealing muscle maintenance until month 5 of the contest preparation. Afterward, muscle thickness measures showed a rapid and severe decline in the last months of preparation. Notably, lean tissue loss coincided with a marked reduction in caloric intake and higher aerobic training volume, whereby the individual achieved extremely low levels of body fat (< 7%). It can be speculated that lean mass loss might heighten when body fat levels fall to a given minimal threshold, as demonstrated in some (Kistler et al. 2014; Robinson et al. 2015) but not all (Mitchell et al. 2018) studies. Higher aerobic training may also interfere with RT adaptations, especially in highly resistance-trained individuals (Petré et al. 2021; Vechin et al. 2021).

The case studies by Robinson et al. (2015) and Kistler et al. (2014) reported large lean mass losses of approximately  – 5.0 kg (~ 43%, skinfolds) and  – 6.6 kg (~ 43%, DXA), respectively. The workout routines included 20–25 sets/workout (Kistler et al. 2014) and 4–5 sets/exercise performed twice per week (Robinson et al. 2015), with no information provided about total tonnage lifted, total sets per muscle group per week or any RT volume increases/decreases over time. Notably, both athletes dieted in a comparably higher deficit with Robinson et al. (2015) equating to  – 882 kcal/day and Kistler et al. (2014) to ~  – 580 kcal/day. Although the study by Robinson et al. (2015) could be categorized as high-volume based on our established qualifications, the extent of lean mass sparing is determined by the interplay of multiple variables (Heymsfield et al. 2011). We propose two possible explanations for the higher observed losses in lean mass in these case studies. First, given the assumption that higher deficits lead to greater lean mass loss (Chaston et al. 2007), we speculate that once the deficit becomes too severe, even anabolic stimuli such as high-volume RT may be unable to counteract diet-induced anabolic resistance (Schoenfeld et al. 2020; Kistler et al. 2014; Robinson et al. 2015; Murphy and Koehler 2020). The total body mass loss exhibited in the examined studies did not necessarily translate into lean mass loss, underscoring total energy deficit as an essential factor in determining lean mass change (Murphy and Koehler 2021). Second, according to the inverted U-shaped relationship of hypertrophy proposed by Schoenfeld et al. (2017b), the individual RT volume could be possibly either not high enough or, alternatively, too high, as RT volume must always be considered in a relative (e.g., compared to the off-season) manner (Scarpelli et al. 2020). If volume is decreased from the off-season to the weight loss phase, as has been previously reported in bodybuilders (Hackett et al. 2013), this conceivably reduces mechanical loading, which in turn may hasten the decrease in lean mass (Hornberger 2011; Vandenburgh et al. 1999; Gao et al. 2018; Breen et al. 2013).

Since important information regarding RT variables often was not reported, calculating total tonnage or sets per muscle group per week was only possible in one study and, hence, precluded us from drawing relevant conclusions in this regard. However, based on the inverted U-shaped relationship between RT volume and muscle hypertrophy, the data appear to suggest that progressively increasing RT volume during CR, either by increasing sets per exercise or the load lifted, might be more effective in ameliorating CR-induced atrophy in male resistance-trained athletes than reducing RT volume. When compared to female athletes, increasing RT volume appears to partially counteract, but does not completely reverse diet-induced perturbations in lean tissue in male athletes. Consequently, studies employing mixed-sex designs (Campbell et al. 2020) could not be taken into account due to reporting only summarized results. Although males and females show a similar RT-induced mTOR activation (Dreyer et al. 2010; Smith et al. 2009), they differ in body composition, muscle phenotype, hormonal actions, and mitochondrial activity (Rosa-Caldwell and Greene 2019; Stapley 2001; Williams et al. 2015), and typically retain more lean mass than their male counterparts during phases of CR. Given that sex differences have already been reported for muscle strength and muscle size in an elderly population (Jones et al. 2021), our findings highlight sex as a significant influencing variable that affects lean tissue change during CR. Thus, we recommend future studies that investigate body composition changes during CR to report sex-differentiated results rather than summarizing results. Although research remains somewhat inconclusive on the topic, it might be speculated that a higher estrogen concentration might contribute to lean mass sparing (Carson and Manolagas 2015; Enns and Tiidus 2010), perhaps due to its anabolic effect on insulin-like growth factor (IGF-1) (Olivieri et al. 2014). This hypothesis warrants further investigation.

### Possible explanatory mechanisms

#### Endocrine factors

Hormones take part in a complex signaling system and are affected by exogenous stimuli such as CR, energy availability (Loucks and Thuma 2003; Mountjoy et al. 2018), and physical activity (de Alcantara Borba et al. 2020; Kraemer and Ratamess 2005). For instance, CR induces endocrine changes that may negatively affect protein balance and ultimately contributes to lean mass loss. In this case, the body conceivably conserves energy for more important physiological processes (Trexler et al. 2014). Since IGF-1 data were not reported in the studies included in this review, we solely focused on testosterone changes during CR.

Testosterone is considered a key anabolic hormone that possesses a variety of ergogenic, anabolic, and anti-catabolic properties (Kraemer et al. 2020). Several authors reported lean mass loss after a chronic drop in testosterone in the absence of RT during CR (Friedl et al. 2000; Karila et al. 2008); alternatively, lean mass sparing is reported with non-significant testosterone changes (Huovinen et al. 2015), suggesting a potential role of testosterone on the extent of lean mass sparing during CR. Dietary interventions, including a high-protein approach, are not able to attenuate the testosterone decline during CR (Henning et al. 2014); rather, the drop seems to be energy-dependent (Karila et al. 2008; Mero et al. 2010; Longland et al. 2016) and must be interpreted in the context of other anabolic hormones such as IGF-1(Sculthorpe et al. 2012). In the context of RT, androgen receptor content correlates with an increase in lean mass and m. vastus lateralis type 1 and 2 CSA, respectively, during eucaloric conditions (Morton et al. 2018).

With respect to the studies compiled in this review, 6 studies investigated testosterone change during energy deficit with mixed results. In a case study involving self-reported “moderate-volume” RT (Rohrig et al. 2017), the female athlete’s testosterone level did not drop during contest preparation. In this case, no lean mass loss was observed. Stratton et al. (2020) reported that testosterone concentration changed significantly by a non-consequential amount of  – 6.6 ng/dL (time-restricted feeding) and  – 1.1 ng/dL (normal daily feeding), respectively, with their participants ultimately increasing CSA during CR; RT load was increased during the course of the study. Moreover, no statistical difference in testosterone change was seen in the first 8 weeks of contest preparation in the study by Mitchell et al. (2018), coinciding with increased RT volume. Contrarily, other research reported that testosterone levels dropped in females (Hulmi et al. 2016), with contradictory lean mass changes reported between DXA and MFBIA, and males (Schoenfeld et al. 2020; Pardue et al. 2017). The CR-induced drop in testosterone levels might negatively affect MAPK/ERK1/2 (Dent et al. 2012; Hamdi and Mutungi 2010), mTOR and Akt signaling, as well as androgen receptor activation (Basualto-Alarcón et al. 2013), and thus possibly have a detrimental effect on protein balance (Rossetti et al. 2017).

Although higher acute hormonal concentrations may enhance the anabolic milieu and hence help to spare lean mass (Pritchard et al. 1999; Kraemer et al. 2020), acute post-exercise hormonal elevations are not necessarily reflected in the MPS response (West et al. 2009). Moreover, methodological difficulties such as measurement timing, circadian rhythm, blood volume changes, and hormonal interactions with binding proteins (Craig et al. 2008; Kraemer et al. 2016) make inter-study comparisons challenging. While mediated by a plethora of variables such as energy deficit, energy availability, and sleep quality, the data compiled in this review may suggest a link between higher volume RT, testosterone-level preservation, and lean mass sparing during CR. Nevertheless, this hypothesis remains speculative and warrants further study.

#### Intracellular pathways

Lean mass changes are determined by the dynamic balance between MPS and protein breakdown (Biolo et al. 1995; Phillips et al. 1997). Termed as protein turnover, this ratio is affected by many variables such as energy and nutrient availability, growth-related hormones, sleep status, and mechanical loading (Areta et al. 2014; Hoppeler 2016; Pasiakos and Carbone 2014). In the perspective of the latter, high-volume RT elicits reactions in metabolic, endocrine, nervous, and musculoskeletal systems (Kraemer and Ratamess 2004).

The mechanistic target of rapamycin (mTOR) functions as a molecular nodal point that modulates the magnitude and duration of MPS (Hoppeler 2016). During CR, activation of mTORC1 and its downstream targets, as well as MPS is reduced, while proteolysis appears to be increased (Margolis et al. 2016; Carbone et al. 2014; Berryman et al. 2017); however, the results in this regard are somewhat conflicting (Carbone et al. 2019). Without any counteracting stimuli (e.g., RT), attenuation of MPS ultimately leads to a negative net protein balance and, hence, to lean tissue loss (Roth et al. 2021; Pasiakos et al. 2013). This is supported by the data of athletes taking performance-enhancing drugs who, due to ergogenic effects on protein turnover, do not show significant lean tissue loss (Pasiakos et al. 2019; de Souza et al. 2018; Howard et al. 2020). Although RT is widely recognized as a potent countermeasure against CR-induced alterations, lean tissue loss is also often reported (Weinheimer et al. 2010).

A change in the protein translation process is believed to be a possible reason for the reduced MPS response to either nutritional or mechanical stimuli during CR, conceivably as an adaptive response by the body to selectively synthesize new proteins depending on cell needs (e.g., surviving function; Margolis et al. 2016; Miller et al. 2012; Carbone et al. 2012). While higher volume RT increases intracellular anabolic signaling during eucaloric conditions (Burd et al. 2010; Terzis et al. 2010; Hulmi et al. 2012; Ahtiainen et al. 2015), similar findings have been reported for CR (Areta et al. 2014). Although resting postabsorptive MPS was reported to be lower during CR (30 kcal/kg^−1^ FFM) when compared to energy balance (Areta et al. 2014), high-volume RT (6 sets × 8 repetitions) elevated MPS to values observed at rest in energy balance, along with further increases after protein supplementation. The study included resistance-trained athletes who undertook a short-term diet (5 days). mTOR^Ser2448^, Akt^Ser473^, p70S6K^Thr389^, and rpS6^Ser236/237^ phosphorylation were restored above resting energy balance baseline levels and no differences were seen in AMPK, 4E-BP1, or eEF2 phosphorylation. Contrarily, lower postabsorptive Akt^Ser473^ phosphorylation as well as lower postprandial p70s6k^Ser424/Thr421^ phosphorylation (similar postprandial p70s6k levels at Thr389) were observed by Pasiakos et al. (2013), who investigated the effect of different protein intakes in physically active individuals undergoing CR. Herein, low-volume and low-intensity resistive type exercise was performed to maintain physical fitness. In contrast to Areta et al. (2014), lower phosphorylation levels of Akt^Ser473^ and p70s6k^Ser424/Thr421^ have been reported. Since CR was comparable in both studies and high-protein approaches were followed, differences in RT volume might be an explanation for the different signaling responses. In addition to RT volume, differences in intensity of effort (proximity to failure) should be considered a potential alternative theory given that proximity to failure might affect hypertrophy in resistance-trained athletes (Grgic et al. 2021).

Although evidence remains preliminary, it appears that multi-set protocols lead to a pronounced upregulation of intracellular anabolic signaling in resistance-trained athletes when compared with low-volume resistive type exercise during CR. However, although intracellular data appear to support the proposed benefits of higher RT volume on lean tissue sparing, further studies are warranted to test this hypothesis.

## Conclusion and limitations

**Fig. 3:**
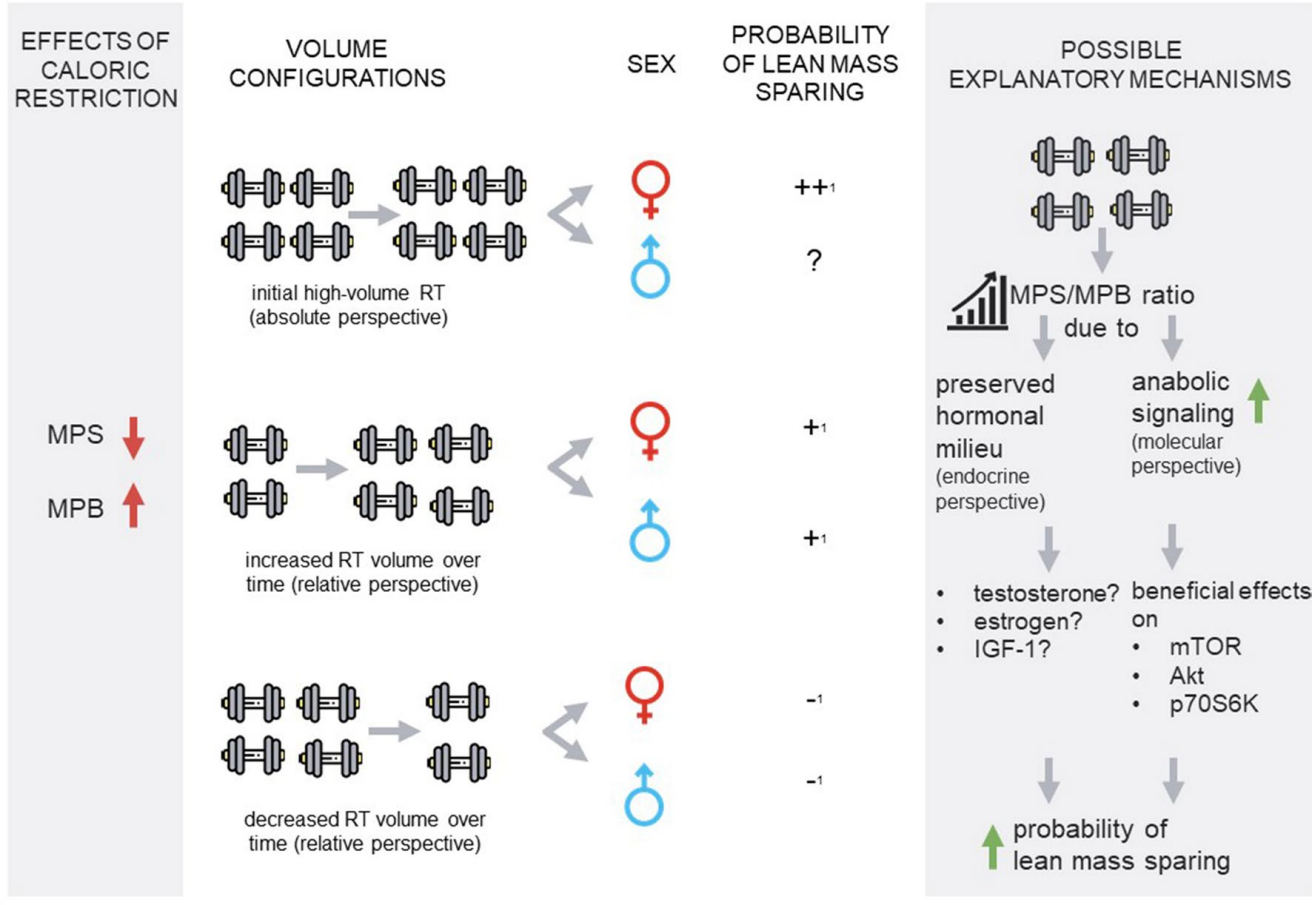
Overview of review findings. ^1^supported by preliminary evidence; plus/minus demonstrates probability of lean mass sparing. This chart was created using images from flaticon.com (Good Ware / Freepik). Green color indicates a beneficial effect on lean mass sparing; red color indicates a detrimental effect on lean mass sparing, *MPS*  muscle protein synthesis, *MPB*  muscle protein breakdown, *RT*  resistance training

The findings of this review suggest that reducing RT volume during CR (van der Ploeg et al. 2001; Vargas-Molina et al. 2020; Mitchell et al. 2018) may negatively affect lean tissue sparing in resistance-trained individuals. Based on the included studies, there is insufficient evidence to conclude that a higher RT volume better spares lean mass during CR, although the available data seem to favor high-volume RT in female athletes under these circumstances. Studies examining resistance-trained males are less conclusive, with 1 study reporting substantial lean mass loss despite being referenced as high-volume; however, the data appear to suggest that systematically increasing volume during CR may enhance the anabolic milieu when compared to reducing RT volume. Hence, increasing resistance training volume might be a promising approach to maximize lean tissue sparing during CR (Fig. 3). Possible effects seem to be mediated by training experience, pre-diet volume, energy deficit, body fat, and concurrent aerobic training. Accordingly, advanced lifters or athletes adapted to high mechanical loading protocols (e.g., powerlifters) might require more volume compared to recreationally trained athletes (Stratton et al. 2020), similar to what has been reported for muscle strength gains (Peterson et al. 2005).

When stratified by sex, our findings also indicate that women tend to retain more lean mass compared to males when using higher volume RT during CR. However, randomized controlled trials on the topic are lacking (Fagerberg 2018; Helms et al. 2015a). Furthermore, methodological differences (e.g., protein amount, RT experience, initial fat mass, or volume quantification), study quality, and the varied assessment techniques (4C, DXA, BIA, ADP, UWW, skinfolds, and sonography) confound the ability to draw strong conclusions. Since data regarding manipulation of RT variables were incomplete in most of the included studies, findings need to be interpreted with caution. Based on the 2791 papers screened, only 15 studies met inclusion criteria and, hence, were included in qualitative analysis. However, where applicable, relevant information from other studies was incorporated into the conclusions drawn herein.

Contrary to our conclusion, athletes are often instructed to reduce volume during phases of CR (Gentil 2015; Chaouachi et al. 2009; Meckel et al. 2008). This practice is typically justified by the claim of higher recovery demands under conditions of low-energy availability and often referenced with the study of Bickel and colleagues (2011), who demonstrated that young, intermediate-experienced athletes can preserve lean mass during eucaloric conditions when training with approximately one-third of their original volume. However, CR is associated with a suppression of anabolic and anti-catabolic stimuli, as well as a heightened catabolic milieu. From an intracellular signaling perspective, findings of eucaloric and hypercaloric studies cannot be extrapolated to hypocaloric conditions due to skeletal muscle probably becoming less sensitive to nutritional and mechanical stimuli during CR (Murphy and Koehler 2020). Consequently, RT volume should not necessarily be decreased during phases of prolonged CR.

It is important to note that our conclusions are based on correlational data, which precludes the ability to draw strong causal inferences. Future research should focus on conducting randomized controlled interventions that directly compare higher versus lower RT volume protocols during periods of CR to better understand the cause–effect relationship between training volume and energy availability.

## Data Availability

Not applicable.
